# 肺癌围手术期肺康复训练中国专家共识

**DOI:** 10.3779/j.issn.1009-3419.2024.102.25

**Published:** 2024-07-20

**Authors:** 中国老年保健协会肺癌专业委员会, 四川省康复医学会加速康复外科分会, 成都康复医学会肺康复专业委员会

**Keywords:** 肺肿瘤, 围手术期肺康复, 专家共识, Lung neoplasms, Perioperative pulmonary rehabilitation, Expert consensus

## Abstract

围手术期肺康复能有效降低术后肺部相关并发症的发生并提高肺癌患者术后的生活质量，在肺癌患者中的临床应用价值已被广泛认可。然而肺康复方案仍然没有形成国际共识和指南，运用于肺癌围手术期临床实践时缺乏规范和标准。本共识将通过提供围手术期肺康复训练的实施方案、流程，促进围手术期肺康复训练在临床实践中更合理、更规范地应用，最终能够使患者最大程度地从中获益。

## Abstract (en)

Perioperative pulmonary rehabilitation may effectively reduce the incidence of postoperative pulmonary complications and improve the quality of life of lung cancer patients and its clinical application value in lung cancer patients has been widely recognized. However, there is still no international consensus or guideline for pulmonary rehabilitation regimen, lacking standardized criteria when pulmonary rehabilitation applied in perioperative clinical practice for lung cancer. The consensus provides implementation regimen and process of pulmonary rehabilitation, aiming to promote the reasonable and standardized application of perioperative pulmonary rehabilitation training in clinical practice, sequentially enable patients to maximize benefits from the rehabilitation.

肺康复作为一种个体化干预方法，可缓解肺癌患者相关症状，改善躯体功能和心理状态，提高生活质量并促进其建立长期的健康行为。常用肺康复干预措施包括但不限于运动训练、气道廓清、呼吸训练、健康教育和行为干预等，其中运动训练是肺康复的核心内容。研究^[1⇓-3]^表明，对于因肺功能差而失去手术机会的肺癌患者，通过术前肺康复训练可获得手术机会；肺癌合并高危因素患者[如高龄、慢性阻塞性肺疾病（chronic obstructive pulmonary disease, COPD）]等，术前肺康复训练可以降低肺癌患者术后肺部相关并发症（postoperative pulmonary complications, PPCs）的发生风险；术前肺功能正常或风险相对较低的肺癌患者，通过肺康复训练可以改善术后生活质量并减轻相关症状（如咳嗽和疲劳等）。肺康复训练运用于肺癌围手术期临床实践时仍有许多障碍，包括不同团队所采取的评估方法、评估标准和实施方案存在差异，肺康复临床效果也大不相同^[4⇓-6]^，导致实施的可操作性和可重复性受到限制等。围绕肺癌围手术期肺康复常见问题，四川大学华西医院肺癌中心联合国内专家起草了《肺癌围手术期肺康复训练中国专家共识》，通过提供围手术期肺康复训练的实施方案、流程，促进围手术期肺康复训练在临床实践中更合理、更规范地应用。

## 1 共识制定方法

本共识基于相关临床研究、系统评价、临床指南等当前最佳证据，并参考《世界卫生组织指南制定手册》（2014年版），通过广泛征求相关专家意见进行修改制定。本共识检索了PubMed、Web of Science、万方、中国知网等数据库近20年的相关研究。基于循证证据和临床经验，专家共识编写组共召开5次会议，各专家间私下交流不做记录。共识编写从2023年1月正式启动，通过大纲讨论、内容撰写、内容讨论及修改、统稿、内容审校、共识全文通读审核，至2024年2月最终定稿。

本共识的推荐级别如下：Ia级：风险与收益明确，基于无缺陷随机对照临床试验结果，强烈推荐，无保留地在各种环境应用于绝大多数患者；Ib级：风险与收益明确，基于有缺陷随机对照临床试验结果，强烈推荐，可应用于绝大多数患者；Ic级：风险与收益明确，基于观察性研究结果，中度推荐，可能因可靠的研究证据出现而改变。

## 2 肺康复临床团队的建立

**推荐1：肺康复团队至少应由胸外科、康复科、护理团队组成，在肺癌手术患者全程治疗的各个阶段制定相应的肺康复训练计划，院内康复和居家康复方案应有所区别（Ic级）。**

肺康复贯穿于肺癌患者住院前、术前、术中、术后及出院后的全治疗过程，需要医护一体化及呼吸科、麻醉科、疼痛科、康复科、营养科等多学科诊疗（multi-disciplinary treatment, MDT），保障肺康复顺利实施。肺康复训练一人一策计划时，多学科团队必须评估患者的疾病状态与康复获益，如考虑康复疗程与肿瘤进展风险等，制定相应强度和时间的康复训练计划。

## 3 肺康复训练的适用人群

**推荐2：肺癌手术患者，尤其是合并高危因素患者，围手术期均应进行个体化的肺康复训练（Ib级）。**

肺癌患者术前身体状态是决定手术风险、手术方式及手术结局的重要因素，尤其是心肺功能。理论上所有肺手术患者均可进行围手术期肺康复训练，临床上通过术前高危因素评估决定最合适的肺康复人群，并由此制定不同的肺康复训练方案^[7⇓-9]^。

## 4 术前危险因素评估

### 4.1 年龄

**推荐3：年龄≥70岁的高龄患者，术前应进行1-2周中-高强度肺康复训练（Ib级）。**

高龄患者心肺功能、运动耐力下降，合并基础疾病多，手术耐受能力、抵御手术创伤能力随之下降，围手术期风险显著增加。但年龄本身并不是手术的禁忌证，对老年患者的心肺功能进行全面评估有助于判断高龄患者手术风险。研究^[10⇓-12]^证实针对高龄（≥70岁）肺癌患者术前实施短期中-高强度肺康复训练可改善心肺功能及运动耐力，降低PPCs发生率，缩短住院时间等。

### 4.2 肺功能评估

**推荐4：肺功能正常或轻度受损的患者，术前可进行1周中-高强度肺康复训练（Ic级）。**

**推荐5：肺功能中-重度及以上受损的患者，术前应进行2-4周中-高强度肺康复训练（Ib级）。**

肺功能是肺癌患者术前评估的必备检查之一，术前肺功能状态与PPCs的发生密切相关。术前肺功能以第一秒用力呼气容积（forced expiratory volume in the first second, FEV_1_）和一氧化碳弥散量（diffusing capacity of the lungs for carbon monoxide, DLCO）为主要判断指标。肺切除术的标准为：FEV_1_>1 L可以耐受肺叶切除术；FEV_1_>2 L可以耐受全肺切除术^[13]^。FEV_1_绝对值的下降提示术后并发症和围手术期死亡风险的增加，但FEV_1_绝对值容易受到多种因素的影响，比如身高、体重、年龄、性别等，因此FEV_1_绝对值并不能充分反映患者的肺功能状态。FEV_1_%和DLCO%（实测值占预计值的百分比）能相对客观地反映患者个体化的肺功能状态，以肺段为单位来计算预计术后肺功能水平可更好地预测PPCs的发生，且预计术后FEV_1_（predicted postoperative FEV_1_, ppoFEV_1_）的应用优于术前肺功能绝对值，并能减少个体间差异，其计算公式为：ppoFEV_1_=术前实测FEV_1_×（1-被切除有功能的肺段数/具有功能的肺段总数）^[14]^。PPCs的发生风险与ppoFEV_1_密切相关，ppoFEV_1 _%=40%可作为判断能否手术的临界值，当ppoFEV_1_%低于40%时，患者PPCs发生率和死亡率均显著升高^[15]^。

目前肺康复已被广泛应用于临床实践，包括气道廓清技术、呼吸训练等，都有助于保持术后气道的通畅，增加吸气肌肉力量、缓解吸气肌肉张力^[16⇓-18]^。研究^[19⇓-21]^表明，术前肺康复训练可改善患者的氧合能力，改善手术带来的呼吸肌力量下降和呼吸功能紊乱，对于所有接受肺切除的患者术前进行为期1周的高强度肺康复训练可降低PPCs发生率并缩短住院时间。Lai等^[22]^的随机对照试验结果显示，对于合并ppoDLCO%<60%等危险因素在内的患者，术前进行1周高强度肺康复训练能显著改善患者的呼气峰流速（peak expiratory flow, PEF）和6分钟步行距离（6-min walking distance, 6-MWD），并降低PPCs的发生率。针对中重度肺功能受损患者，通常推荐2-4周术前肺康复训练^[23,24]^，并应在训练完成后再次评估心肺功能和手术风险。因此，针对接受肺部手术的患者均可行术前肺康复训练；而对于有肺功能受损的患者，应根据肺功能结果评估肺功能受损程度并制定相应肺康复训练方案，从而改善患者的呼吸功能和氧合能力，最终降低手术风险及PPCs发生率。

### 4.3 运动耐量评估

**推荐6：拟行肺癌手术患者，术前应进行运动耐量评估（Ia级）。**

**推荐7：如患者行往返步行试验（shuttle walk test, SWT），步行>400 m或登楼梯实验高度>22 m，或CPET结果显示VO**_**2**_ **max>20 mL/（kg·min）或者75%预计值，术前应进行1周短期中-高强度肺康复训练（Ic级）。**

**推荐8：如患者行SWT，步行<400 m或登楼梯实验高度<22 m，或心肺运动试验（cardiopulmonary exercise testing, CPET）结果显示最大摄氧量（maximum oxygen uptake, VO**_**2**_ **max）为10-20 mL/（kg·min）或者35%-75%预计值，术前应进行2周中-高强度肺康复训练（Ib级）。**

**推荐9：如患者CPET结果显示VO**_**2**_ **max<10 mL/（kg·min）或者<35%预计值，应进行2-4周术前中-高强度肺康复训练后再次评估手术风险（Ib级）。**

运动耐量评估的目的在于识别静息状态下所不能发现的功能受限及高危风险人群，进而指导临床早期干预措施，简易评估方法主要包括6分钟步行测试（6-min walking test, 6-MWT）、SWT、爬楼梯试验（stair climb test, SCT）。这类方法对场地和设备要求低，操作简单、方便易行，受试者活动量接近日常活动，配合度及满意度较高，但不能更客观精准地反映患者有氧耐受能力^[25⇓⇓-28]^。CPET得出的指标被认为是评价肺切除手术风险和量化评估心肺功能的“金标准”，与简易运动测试相比，CPET可在受控制的环境中连续监测各种呼吸、心脏及代谢参数，是一个标准化运动测试，具有良好的可重复性，可准确地识别氧转运系统中的各种问题，量化手术干预或临床治疗效果，指导手术方案及个体化运动处方的制定^[29⇓-31]^。

美国胸科医师学会（American College of Chest Physicians, ACCP）肺癌指南指出如患者行SWT评估，步行<400 m或登楼梯实验高度<22 m提示围手术期手术死亡和心肺相关并发症发生风险增加^[6]^；而在CPET评估方面，研究^[30,32⇓-34]^认为VO_2_max为10-20 mL/（kg·min）或者35%-75%预计值提示手术风险增加，VO_2_max<10 mL/（kg·min）或者<35%预计值认为是手术高危因素。研究^[32,35⇓⇓-38]^表明，通过术前肺康复训练可有效提高患者6-MWD、VO_2_max等运动功能指标，改善患者运动耐受能力，降低PPCs发生率。既往研究^[36⇓-38]^中术前肺康复训练多在门诊或社区医院实施，训练时间为2-6周，训练频率和强度相对较低，近年来研究^[10,11,22]^已证实术前短期高强度肺康复训练可有效改善肺癌手术患者心肺功能，降低PPCs发生率，缩短平均住院时间。此外，针对手术高风险人群，可进行2-4周术前肺康复训练后再次评估手术风险^[30,39]^。

### 4.4 营养状况

**推荐10：对于身体质量指数（body mass index, BMI）>28 kg/m** ^**2**^ **或<18 kg/m** ^**2**^ **的超重/体重过轻患者，术前应进行1周及以上中-高强度肺康复训练（Ic级）。**

良好的营养状况是维持机体正常生命活动的重要保证。无论是营养不良还是肥胖都是手术的危险因素^[11,12]^。临床上常用BMI来评价患者营养状况。肥胖患者的病理生理改变可导致其呼吸储备功能和运动耐力下降，氧耗量和二氧化碳产生量增加，同时合并心血管、呼吸系统疾病风险升高；研究^[40,41]^表明BMI>28 kg/m^2^或<18 kg/m^2^显著增加肺癌术后肺部感染风险。Lai等^[22]^研究表明针对肥胖患者，除了早期营养干预和饮食指导外，通过术前1周短期高强度肺康复训练可有效提高其心肺功能及运动耐力，降低PPCs的发生风险。

### 4.5 戒烟时间及吸烟指数

**推荐11：术前至少戒烟时间2周，推荐4周（Ib级）。**

**推荐12：吸烟指数≥400年支且/或戒烟时间不足2周患者，术前应进行至少1周中-高强度肺康复训练（Ib级）。**

吸烟是诱发肺癌的重要危险因素，并显著增加PPCs发生率和围手术期死亡风险^[42⇓⇓⇓-46]^。大量证据^[44⇓-46]^表明，吸烟患者术后PPCs发生率高于从未吸烟者。吸烟量一般使用吸烟指数表示（每日吸烟数×吸烟年数），吸烟指数≤200年支为轻度吸烟，200-400年支为中度吸烟，≥400年支为重度吸烟^[47]^。目前国内普遍认为有吸烟史的患者术前至少应戒烟2周以上，更多的研究^[48⇓⇓-51]^则认为在不影响疾病进展的情况下建议术前戒烟4周以上，可以明显降低术后并发症和气道炎症水平。沈春辉等^[52]^根据肺癌患者不同的吸烟指数制定个性化的肺康复方案，可以显著提高肺癌患者的运动耐力。吸烟患者在完成2周肺康复训练（呼吸肌训练+有氧训练）基础上，进行祛痰、平喘等药物干预。Gao等^[53]^对吸烟患者使用同样的肺康复方案，肺康复时间缩短为3-7 d，肺康复组PPCs发生率和术后住院日仍有显著降低。此外，Lai等^[22]^发现通过1周高强度肺康复训练，吸烟>20年包（400年支）的患者临床明显获益。因此，针对合并吸烟这一危险因素的患者，可推荐术前1周及以上中-高强度肺康复训练，必要时联合药物进行气道管理。

### 4.6 呼吸肌功能评估

**推荐13：最大吸气压（maximal inspiratory pressure, MIP）、最大呼气压（maximum expiratory pressure, MEP）低于80 cmH**_**2**_ **O的患者，术前可行至少2周中-高强度肺康复训练（Ic级）。**

胸外科手术导致的呼吸肌损伤、术后疼痛和卧床制动使术后咳痰能力降低，膈肌活动能力不足，是引起术后肺不张和肺炎的重要因素之一^[54]^。MIP与MEP可作为评估呼吸肌肌力的重要指标。术前MIP与MEP较低的患者，术后MIP与MEP的下降更明显；开胸手术对呼吸肌的影响更为显著，尽管术后短时间内会有部分肌力恢复，但12周之内都仍低于术前水平。术前MIP、MEP低于80 cmH_2_O呈现PPCs发生率增加的趋势，且随着年龄的增加PPCs的发生风险增加。研究^[54⇓⇓⇓-58]^表明通过术前吸气肌肌力训练可有效提升MIP、MEP水平，改善心肺功能，从而降低PPCs发生率并缩短住院时间。

### 4.7 新辅助治疗或肺部二次手术

**推荐14：新辅助治疗或肺部二次手术患者的肺癌患者，术前应进行至少1-2周肺康复训练（Ic级）。**

对于局部晚期肺癌患者，新辅助治疗可最大程度提高近、远期生存率，使患者最大生存获益。然而新辅助治疗如放化疗、免疫治疗等，可使患者运动耐力下降、心肺功能受损，加重呼吸及心血管系统症状^[59]^。研究^[60,61]^显示，针对肺癌患者在放化疗期间实施肺康复训练可有效改善呼吸功能，且在新辅助治疗期间通过肺康复训练以维持和改善患者心肺功能具有重要临床意义。

既往已行肺部手术的患者，术中及术后均会造成不同程度的肺损伤和肺功能受损，具体包括手术中单肺通气和术中高气道压，可同时损伤通气侧和非通气侧肺组织，且损伤程度随时间延长而增加^[62]^；以及肺切除术后肺损伤的“双时相”表现，包括原发性和继发性肺损伤等^[63,64]^。因此，在进行肺部二次手术时，应考虑到手术对患者肺功能的进一步损害，充分评估患者术前心肺功能，判断其能否耐受再次手术。围手术期肺康复训练有益于患者对肺部二次手术的耐受，降低PPCs发生风险，改善其远期生活质量^[8,9,65,66]^。目前有关肺部二次手术患者进行围手术期肺康复训练相关证据较少，但是基于二次手术对患者潜在的不利影响，仍然可以考虑进行术前1-2周的肺康复训练。

## 5 术前肺康复训练方案

### 5.1 运动训练

**推荐15：肺癌术前肺康复训练建议以有氧训练、抗阻训练及呼吸肌肌力训练相结合的多模式综合运动训练为主，高强度训练1-2周，中强度训练2-4周（Ib级）。**

研究^[67]^显示术前运动训练可使肺癌患者术后并发症降低48%，住院时间缩短3 d。运动训练不但可以提高肺通气功能、优化肺通气/血流（V/Q）比值，还能增加心脏的心肌收缩能力、增强全身骨骼肌功能并提升肌肉的摄氧能力等^[56⇓-58]^。其中，有氧训练、全身骨骼肌抗阻训练及呼吸肌力量训练是运动训练的主要内容。有氧训练方式包括快走或慢跑、功率自行车训练等；训练强度推荐为中-高强度训练；持续时间一般为30-60 min；频率为3-5次/周^[22,37,67,68]^。抗阻训练方案中，应以大肌肉群的力量训练为主，训练方式可以是哑铃、弹力带、依靠自身重量的抗阻训练以及其他利用器械的抗阻训练；训练强度为一次可重复最大重量（one repetition maximum, 1RM）的40%-60%，每个肌群8-12次/组，2-3组/次、频率为2-3次/周^[57,69]^。呼吸肌肌力训练方式可采用呼吸肌训练器，吸气肌肌力训练强度一般为10%-40%的MIP，呼气肌肌力训练强度为4-20 cmH_2_O，15 min/次，训练疗程推荐>2周^[19,70]^。

术前运动训练疗程为1-4周。临床实践中可根据训练强度、患者心肺功能程度来决定具体训练疗程：高强度1-2周，中强度2-4周，不建议超过8周。此外，研究^[38,71⇓-73]^显示有氧训练、抗阻训练及呼吸肌肌力训练的综合运动训练方案比单一的运动训练方式在改善患者心肺功能上更具备优势。

### 5.2 气道廓清技术

**推荐16：肺癌手术患者围手术期应采取气道廓清技术，时间为1-2周（Ic级）。**

合并COPD的肺癌患者气道分泌物增多且易造成滞留。肺部手术后的急性炎症反应使气道分泌物显著增多、气道阻力增大且使分泌物排出困难，导致PPCs发生率增加。对于这部分患者应根据患者的情况，结合药物与气道廓清技术，促进呼吸道内分泌物的清除，减少患者呼吸相关症状，降低PPCs的发生率^[32,39,74,75]^。气道廓清技术类型包括主动和辅助咳嗽训练、主动循环呼吸技术、自主引流技术、呼气正压振动排痰仪等^[75,76]^。

## 6 术后肺康复方案

### 6.1 早期活动

**推荐17：肺癌术后患者应尽量在术后24 h内下床活动（Ib级）。**

术后卧床制动可造成患者身体机能失调，血液循环能力降低，肌肉量减少，肺通气降低，导致术后肺不张、肺部感染、肺栓塞等PPCs的发生风险增加。早期活动（24 h之内）不仅能降低卧床制动带来的不利影响，还能减轻术后疼痛、减少管道滞留对呼吸的消极影响，从而降低PPCs的发生率，缩短住院时间^[9,77,78]^。

### 6.2 术后运动训练

**推荐18：肺癌患者术后2周，若无伤口或其他严重并发症，可评估并制定肺康复训练计划，训练时间为4-12周（Ib级）。**

肺癌患者术后出现的呼吸困难、咳嗽、疲劳、运动能力下降、睡眠障碍等问题很大程度上影响了患者的生活质量^[79,80]^。已有研究^[80]^证实术后坚持长期运动训练的患者生活质量显著提高，术后的肺康复训练方式以运动训练为主，主要结合心肺有氧训练、全身骨骼肌大肌群的抗阻训练、柔韧性训练、呼吸肌肌力训练等。术后训练开始的时间及训练方案存在差异，介入时间主要集中在术后2-4周，训练疗程在4-12周。术后训练方案实施需根据患者术后康复情况、对伤口或术后并发症的影响进行及时调整^[80⇓-82]^。

## 7 结语

肺癌围手术期肺康复训练应根据循证医学证据实施，目前应用的围手术期肺康复训练方案仍有很大局限性，但临床应用的效果较好。本共识专家团队依据近年来新的临床研究证据和临床实践，制定了《肺癌围手术期肺康复训练中国专家共识》，以期能推动肺癌围手术期肺康复训练在临床中更好地实施和推广，推进临床多中心研究，获取更多的临床研究数据，使得肺癌围手术期肺康复训练方案在临床实践中更加完善，从而服务于更多的患者。

**Table:** 

肺癌围手术期肺康复训练专家共识编写组	
**组长**	
车国卫	四川大学华西医院
周清华	四川大学华西医院
**执笔人**	
赖玉田	四川大学华西医院
王娇	四川大学华西医院
李晓鸥	四川大学华西医院
喻鹏铭	四川大学华西医院
**编写组成员**（按姓氏汉语拼音排序，排名不分先后）	
陈军	天津医科大学总医院
车国卫	四川大学华西医院
陈曦	四川大学华西医院
戴维	四川省肿瘤医院
付军科	西安交通大学第一附属医院
范军强	浙江大学医学院附属第二医院
高珂	成都市第二人民医院
高强	四川大学华西医院
郭占林	内蒙古医科大学附属医院
蒋丽莎	四川大学华西医院
林嵘嘉	福建省立医院
龙谦	贵州省人民医院
赖玉田	四川大学华西医院
李书平	成都医学院第一附属医院
李晓鸥	四川大学华西医院
李洪娟	四川大学华西医院
乔坤	深圳市第三人民医院
苏建华	四川大学华西医院
苏巧俐	四川大学华西医院
屠政良	浙江大学医学院附属第一医院
韦海涛	河南大学淮河医院
吴齐飞	西安交通大学第一附属医院
魏全	四川大学华西医院
王娇	四川大学华西医院
王一帆	成都大学附属医院
王毅	内江市第一人民医院
王维	四川大学华西医院
王继勇	广州中医药大学附属一医院
谢薇	四川大学华西医院
薛涛	东南大学中大医院
喻鹏铭	四川大学华西医院
叶芸	四川大学华西医院
曾富春	四川省人民医院
张鹏	天津医科大学总医院
周清华	四川大学华西医院
周坤	浙江大学医学院附属第一医院
